# Personal and clinical traits in adolescents, diagnosed with «anorexia nervosa»

**DOI:** 10.1192/j.eurpsy.2021.953

**Published:** 2021-08-13

**Authors:** E. Balakireva, N. Zvereva, S. Voronova

**Affiliations:** 1 Child Psychiatry, Federal State Budgetary Scientific Institution Mental Health Research Center, Moscow, Russian Federation; 2 Clinical Psychology, Federal State Budgetary Scientific Institution Mental Health Research Center, Moscow, Russian Federation

**Keywords:** anorexia nervosa, adolescents, eating disorders

## Abstract

**Introduction:**

Eating disorders are among the most common mental health problems. The prevalence of diseases in this circle is 1-2% of the population; in adolescents - 1%. There is a significant “rejuvenation” of eating disorders with the appearance anorexia nervosa cases in preschool and primary school age. The prevalence of such disorders among adolescents is a significant reason for detailed and comprehensive study of the issue. Many factors lead to development of eating disorders: genetic predisposition, family background, socio-cultural factors, life experience. We suggested that due to many mutually overlapping factors in the syndrome of anorexia nervosa, there may also be distortions of personality characteristics, sometimes reaching the level of personality disorders.

**Objectives:**

assessment of personality feachers 34 patients with leading diagnosis of F-50.0 (ICD-10) were examined in FSBSI MHRC (inpatient treatment/outpatient observation). All adolescents received drug therapy.

**Methods:**

The study was carried out using modern pathopsychological methods with the inclusion of research questionnaires aimed at identifying personal pathology (LoPF 12-18, AIDA).

**Results:**

During the research, the following personality traits were revealed: perfectionism, the desire to correspond to a certain ideal image of oneself, instability of Ego, unstable identity violations; reduced ability to form a picture of the future and themselves in the future; also showed a tendency to abuse psychoactive substances.

**Conclusions:**

Thus, the general for all patients with diagnosed disorder was persistent refusal to eat (up to dystrophy), distortion of Ego, characteristic of personality disorders were also observed. Further studies are required to obtain a more detailed picture and clarify the prognostic outcome.

